# Host-Guest Complexation of Oxaliplatin and *Para-*Sulfonatocalix[n]Arenes for Potential Use in Cancer Therapy

**DOI:** 10.3390/molecules25245926

**Published:** 2020-12-14

**Authors:** Sherif Ashraf Fahmy, Fortuna Ponte, Iten M. Fawzy, Emilia Sicilia, Udo Bakowsky, Hassan Mohamed El-Said Azzazy

**Affiliations:** 1 Department of Chemistry, School of Sciences & Engineering, The American University in Cairo, AUC Avenue, P.O. Box 74, New Cairo 11835, Egypt; sheriffahmy@aucegypt.edu; 2 Department of Chemistry and Chemical Technologies, University of Calabria, 87036 Arcavacata di Rende, Italy; fortuna.ponte@unical.it (F.P.); Emilia.sicilia@unical.it (E.S.); 3 Pharmaceutical Chemistry Department, Faculty of Pharmaceutical Sciences and Pharmaceutical Industries, Future University in Egypt, Cairo 12311, Egypt; iten.mamdouh@fue.edu.eg; 4 Department of Pharmaceutics and Biopharmaceutics, University of Marburg, Robert-Koch-Str. 4, 35037 Marburg, Germany

**Keywords:** oxaliplatin, 4-sulfonatocalix[4]arenes, 4-sulfonatocalix[6]arenes, host-guest complex, cancer therapy

## Abstract

*P*-sulfonatocalix[n]arenes have demonstrated a great potential for encapsulation of therapeutic drugs via host-guest complexation to improve solubility, stability, and bioavailability of encapsulated drugs. In this work, guest-host complexes of a third-generation anticancer drug (oxaliplatin) and *p*-4-sulfocalix[n]arenes (*n* = 4 and 6; *p*-SC4 and *p*-SC6, respectively) were prepared and investigated, using ^1^H NMR, UV, Job’s plot analysis, and DFT calculations, for use as cancer therapeutics. The peak amplitude of the prepared host-guest complexes was linearly proportional to the concentration of oxaliplatin in the range of 1.0 × 10^−5^ M^−1^ to 2.1 × 10^−4^ M^−1^. The reaction stoichiometry between either *p*-SC4 or *p*-SC6 and oxaliplatin in the formed complexes was 1:1. The stability constants for the complexes were 5.07 × 10^4^ M^−1^ and 6.3 × 10^4^ M^−1^. These correspond to complexation free energy of −6.39 and −6.52 kcal/mol for *p*-SC4 and *p*-SC6, respectively. Complexation between oxaliplatin and *p*-SC4 or *p*-SC6 was found to involve hydrogen bonds. Both complexes exhibited enhanced biological and high cytotoxic activities against HT-29 colorectal cells and MCF-7 breast adenocarcinoma compared to free oxaliplatin, which warrants further investigation for cancer therapy.

## 1. Introduction

**Figure 1:**
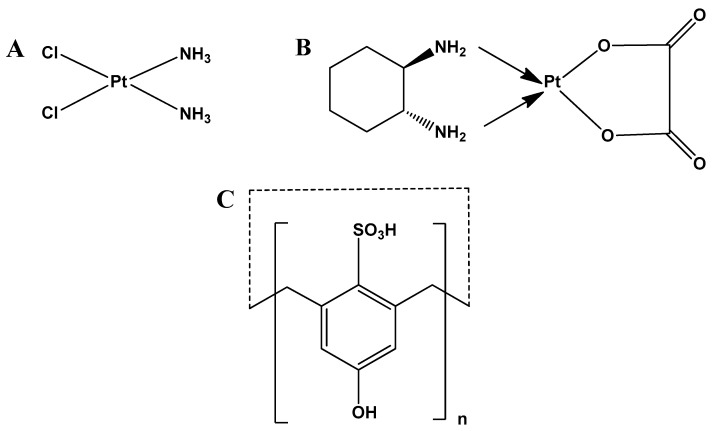
Chemical structures of (**A**) cisplatin; (**B**) oxaliplatin; (**C**) *p*-SC4 (*n* = 4), and *p*-SC6 (*n* = 6).

Platinum drug-based chemotherapeutics (PBCs) are among the most potent broad-spectrum anticancer medicines which are used in chemotherapy of 50–70% of cancer patients [1,2]. The cytotoxic effects of cisplatin, the first generation PBC, were discovered coincidentally by Barnett Rosenberg in 1965 and found to possess potent broad-spectrum anticancer activity among different types of solid tumors [3,4]. Since the approval of cisplatin by the US Food and Drug Administration (FDA) in 1978, thousands of PBCs have been developed and tested in clinical trials to achieve chemotherapeutic agents with higher anticancer activities and fewer side effects compared to cisplatin. Only oxaliplatin and carboplatin were granted FDA approval for cancer treatment [5]. Oxaliplatin [trans-R, R-1,2-diaminocyclohexane)oxalatoplatinum(II)] is a revolutionary third-generation platinum derivative that has been developed to overcome the limitations of cisplatin [6,7]. In oxaliplatin, the two amine carrier ligands in cisplatin are replaced with the lipophilic 1, 2-diaminocyclohexane (DACH) carrier ligand. The two chloride leaving groups in cisplatin are also replaced with the more inert oxalate ligand that binds to the central platinum metal in a bidentate mode (Figure 1A,B) [6,7,8]. The mode of cytotoxicity of oxaliplatin is similar to that of cisplatin and proposed to involve two stepwise hydrolysis steps resulting in the removal of oxalate ligand and the formation of the activated drug species. Oxaliplatin exerts its antitumor activity through the interaction of its activated species with DNA, forming Pt-DNA adducts. There have been studies that proved the role of oxaliplatin in DNA damage of cancerous cells. Molecular modeling studies showed that the 1,2-diaminocyclohexane ring in the oxaliplatin protrudes outward and fills the space of the narrowed major groove of the bound DNA, leading to alteration and formation of a less polar major groove. On the other hand, it was also found that oxaliplatin interacts with ribonuclease (RNase A) through binding to Met 29 and also the formation of bidentate coordination adduct with the RNase A via OD1 atom of Asp 14 [9,10]. Additionally, a recent computational study reported oxaliplatin interaction with insulin, which might affect its cellular uptake into cancerous cells and be responsible for drug resistance. The study revealed the simultaneous binding of two Pt moieties, [Pt(dach)]^2+^ and [Pt(dach)(OH)]^+^ (where dach is 1*R*,2*R*-diaminocyclohexane) to insulin protein host, which gives insight into possible conformational changes undergone by platinum-based drugs due to binding to host proteins under physiological conditions [11].

Oxaliplatin has been developed to overcome cisplatin’s severe adverse effects, including emetogenesis and nephrotoxicity, and intrinsic and acquired resistance [12,13]. Oxaliplatin is the first platinum derivative that was granted FDA approval in the treatment of colorectal cancer, which showed intrinsic resistance to cisplatin [11].

Additionally, oxaliplatin is employed as replacement therapy in relapsed non-Hodgkin’s lymphoma, refractory ovarian cancer, non-small cell lung carcinoma, metastatic breast cancer, and refractory germ cells cancer, when cisplatin treatment fails [14,15]. However, oxaliplatin still suffers from drawbacks including dose-limiting neurotoxicity and accumulations in erythrocytes [15,16]. Besides, several clinical trials showed that oxaliplatin should be used in combination with other anticancer agents for higher therapeutic effects in some resistant cancers [15,16]. Although oxaliplatin is designed to reduce some of the toxic adverse reactions of cisplatin, it still exhibits a remarkable reactivity toward thiol groups that are abundant in the plasma proteins, causing toxic effects [17]. Hence, considerable energies have been exerted to develop novel approaches for the hope of advancing chemotherapy and PBCs in particular. Reformulation of PBCs using different delivery systems is a promising strategy that may enhance their uptake into target cancer cells while simultaneously providing a shield that hinders the deactivation of these chemotherapeutic agents by non-specific reactions in the bloodstream [7]. Several types of delivery systems are reported to encapsulate PBCs [18,19,20,21,22,23]. More recently, supramolecular systems (macrocycles), including calix[n]arenes (CXs), cyclodextrins (CDs), cucurbiturils (CBs), and pillararenes, have attracted attention as possible vehicles for PBCs via either host-guest complexation or self-assembly [24,25,26]. Some previous studies demonstrated the potential use of supramolecular systems as possible carriers for PBCs aiming at enhancing their water solubility, chemical stability, and bioavailability [26]. Additionally, host-guest complexation could also improve the targeting of anticancer drugs to cancer cells resulting in boosting anticancer activities with diminished toxic side effects [26,27,28,29,30]. Moreover, [31,32,33]. CXs, in particular, have attracted much attention during the past decade as significant host molecules that have been used in many fields of supramolecular chemistry. CXs (*n* = 4, 6, and 8) are cone-shaped cyclic oligomers, made up of phenol units connected by methylene bridges. CXs are perfect host molecules for a wide variety of therapeutic guest molecules due to their exceptional structure, which encompasses an upper rim with para-substituent of a phenolic ring, a lower rim with a phenolic hydroxyl group, and a hydrophobic π electron-rich core cavity. The water solubility of CXs is improved by the attachment of some functional groups, including sulfonates and carboxylates at the *para*-position of their phenolic units [34,35,36,37,38,39,40,41,42,43,44,45,46,47,48,49]. Among the various functionalized CX derivatives, the *para*-sulfonatocalix[n]arenes [n = 4 and 6, (*p*-SC4 and-SC6)] demonstrated significant water solubility (>0.1 mol/L), safety, and outstanding biocompatibility to human cells where they show negligible adverse effects at in vivo doses up to 100 mg/kg (Figure 1C,D). Moreover, in aqueous media, *para*-sulfonatocalix[n]arenes were used to accommodate various guest molecules in their cavities [50,51].

Few studies reported that the host-guest complexation between oxaliplatin and host macromolecules might reduce systemic side effects and improve the complex’s antitumor activity compared to the free drug. For instance, the host-guest complexation between oxaliplatin and cucurbit [7] uril (CB7) resulted in the reduction of oxaliplatin’s toxic effects and the enhancement of its anticancer activity against leukemia cancer cells (L1210FR) at significantly lower concentrations compared to free oxaliplatin [26]. In another study, a host-guest complexation between oxaliplatin and CB7 (oxaliplatin-CB7) was found to reduce the cytotoxicity of the complexed oxaliplatin to healthy colorectal cells (NCM460) while enhancing the anticancer activity against cancerous colorectal cells (HCT116, HT29), compared to free oxaliplatin. These effects were attributed to the replacement of oxaliplatin from the complex by spermine, which is overexpressed in cancerous media [26].

This work studies the complexation between either *p*-SC4 or *p*-SC6 and oxaliplatin in aqueous media for possible use in cancer therapy. The formed complexes have been characterized by UV, ^1^H NMR spectroscopy, and computational studies. The stoichiometry and binding constants were detected employing Job’s plot (continuous variation method). The derivative ratio method was implemented to remove the overlapped host spectra and hence measure the UV spectrum of the complex without host interference. Molecular docking and density functional theory (DFT) calculations were implemented to characterize the structure of host-guest complexes. Moreover, the anticancer activities of both complexes against colorectal cancer cells (HT-29) and breast adenocarcinoma (MCF-7) were evaluated using the SRB assay.

## 2. Experimental and Computational Studies

### 2.1. Chemicals and Reagents

Oxaliplatin and 4-sulfocalix [4] arene, *p*-SC4, were obtained from BLD Pharmatech Co. Limited, Cincinnati, OH, USA. 4-sulfocalix [6] arene, *p*-SC6, was purchased from WuXiLabNetwork, Cambridge, MA, USA. Deuterium oxide was purchased from Sigma-Aldrich. Streptomycin, penicillin, fetal bovine serum, trichloroacetic acid (TCA), Dulbecco’s Modified Eagle’s Medium (DMEM) SRB, and tris(hydroxymethyl)aminomethane (TRIS) were purchased from Lonza, Basel, Switzerland.

### 2.2. Instrumentation

UV spectrophotometric measurements were carried out on a CARY 500 UV–Vis-NIR scan dual-beam spectrophotometer (Varian, Palo Alto, CA, USA). ^1^H NMR spectra were measured on a 400 MHz NMR Varian Mercury console™ spectrometer.

### 2.3. Construction of Calibration Graphs (DD^1^ Method)

The zero-order spectra of the prepared calibration solutions were measured and divided by the spectrum of either 0.2 mM *p*-SC4 or 0.2 mM *p*-SC6. The first derivative of the ratio spectra (DD1) was calculated using a scaling factor of 10 and Δλ = 4 nm and its peak amplitudes measured at 267 nm. Calibration curves relating the peak amplitudes of 1DD267 to the corresponding concentrations of oxaliplatin were constructed and the corresponding regression equations were computed.

### 2.4. Cell Viability Assay

Colorectal cancer cells (HT-29) and MCF-7 breast adenocarcinoma (American Type Culture Collection, University Boulevard, Manassas, VA, USA) were maintained in DMEM medium supplemented with streptomycin (100 mg/mL), penicillin (100 units/mL), and 10% heat-inactivated fetal bovine serum. Cells were incubated in 5% (*v*/*v*) CO_2_ at 37 °C.

#### Sulforhodamine B Colorimetric Assay (SRB)

To examine the in vitro anticancer activity of the two complexes, colorectal cancer cell line (HT-29) and MCF-7 breast adenocarcinoma were treated with different concentrations of *p*-SC4, *p*-SC6, oxaliplatin, *p*-SC4-oxaliplatin and *p*-SC6-oxaliplatin complexes. The cell viability of HT-29 cells was evaluated using SRB assay [52,53,54]. Briefly, aliquots of 100 μL cell suspension (5 × 10^3^ cells) were seeded in 96-well plates and incubated in DMEM medium for 24 h at 37 °C and 7% CO_2_. Cells were treated with another aliquot of 100 μL medium containing different concentrations of *p*-SC4, *p*-SC6, oxaliplatin, *p*-SC4-oxaliplatin and *p*-SC6-oxaliplatin complexes (0.01, 0.03, 0.1, 0.3, 1, 3, 10, 30, 100, and 300 μg/mL). After 72 h, culture media were removed and 10% TCA (150 μL) was added at 4 °C for 1 h. The cells were then washed several times with distilled water. SRB solution (70 μL; 0.4% *w/v*) was added and incubated for 10 min (dark at room temperature). Plates were washed three times with 1% acetic acid and permitted to dry overnight. Then, the protein-bound SRB stain was dissolved by adding 150 μL of 10 mM TRIS and absorbance measured at 540 nm (FLUOstar Omega, Ortenberg, Germany).

### 2.5. Computational Details

All the structures were optimized by using the Gaussian 09 program code, [55] at density-functional theory (DFT) level. The B97-D exchange and correlation functional was used[56] in conjunction with the double-zeta 6-31G(d,p) basis set for all atoms except for the oxygen, for which a diffuse function was included and Pt atom, which was described by the Stuttgart/Dresden pseudopotential and the corresponding split valence basis set [57]. This computational protocol was successfully applied for describing similar systems [58]. The aqueous environment was taken into consideration by using the Tomasi’s implicit polarizable continuum model (PCM) [59] as implemented in Gaussian 09, setting the dielectric constant ε at 78.4. The solvation Gibbs free energies were calculated at the same level, performing single-point calculations on gas-phase optimized structures. Vibrational frequency analysis was carried out to confirm the minimum character of the intercepted structures. The Gibbs free energies for the inclusion of the guest (***G***) into calixarene hosts (***H***), in implicit water, ∆Gsol, were calculated as the sum of two contributions: gas-phase free energy **∆*G_gas_*** and a solvation free energy **∆*G_solv_***:

$$∆Gsol=∆G_{gas}+\;∆G_{solv}^{H-G\;complex}-\left(∆G_{solv}^{calix}+∆G_{solv}^{Oxaliplatin}\right)$$

where

$$∆G_{gas}=\;G_{gas}^{H-G\;complex}-\left(G_{gas}^{Oxaliplatin}+G_{gas}^{calix}\right).$$

The binding energies were calculated including the corrections for the basis set superposition error (BSSE) estimated by using the Boys-Bernardi counterpoise technique.[60] The inner cavity volume was estimated by using the Swiss-Pdb Viewer program [61] to evaluate the potential inclusion of oxaliplatin into the host cavities. ADMET study was also performed using Discovery Studio 4.0 to predict the biological properties of both complexes. The complexes possessing the largest interaction energy and best binding mode between oxaliplatin and each of *p*-sulfunatocalix [n] arenes (n = 4 and 6) were then chosen for the energy calculations and ADMET studies. Bulk solvent effects were taken into consideration through minimization using the CHARMm force field and implicit salvation method (GB Generalized Born) in Discovery Studio 4.0, where the implicit solvent model was set with water dielectric environment (ε = 78.39) [56].

## 3. Results

### 3.1. H NMR Spectroscopy

^1^H NMR measurements conducted in D_2_O were performed to examine the complexation between oxaliplatin and *p*-SC4 or *p*-SC6. Figure 1 demonstrates the ^1^H NMR spectra for each of *p*-SC4, oxaliplatin, and 1:1 molar ratio mixture of *p*-SC4 and oxaliplatin (Figure S1A–C respectively). The ^1^H NMR findings revealed that the protons (axial/equatorial) of oxaliplatin cyclohexyl ring have displayed noticeable upfield shifts upon adding *p*-SC4 (Figure S1; Table 1) relative to those of the free oxaliplatin, indicating that the cyclohexyl ring is now located inside the cavity. Possibly this could be explained due to shielding of the aromatic wall of *p*-SC4, where previous studies reported the direct relationship between the extent of the chemical shift of the protons of a guest molecule and their degree of penetration inside the hollow cavity of the host molecule [32,62,63]. This shows that if the oxaliplatin is embedded inside *p*-SC4, it would be embedded via its cyclohexane ring into the *p*-SC4 cavity. Additionally, it was observed that the signal of the methylene bridges protons, Hy, of *p*-SC4, shifted upon complexation with oxaliplatin (0.16 ppm) from 3.67 ppm to 3.83 ppm with a noticeable widening compared to the same signal in *p*-SC4 alone (Figure S1A). This significant signal widening indicates the conformational rigidity of the supramolecular host structure induced by its complexation with the guest molecule [31,64,65]. The structure of *p*-SC4 is flexible which allows the formation of inclusion complexes. The conformational rigidity of *p*-SC4 is achieved when the inclusion complex is formed due to the interactions that are established with the guest. The interactions block the degrees of freedom of the host and rigidity of the cavity is induced. The complexation of oxaliplatin with *p*-SC6 exhibited very similar behavior, suggesting the formation of stable host-guest interactions.

### 3.2. UV-Vis Spectroscopy

**Figure 2:**
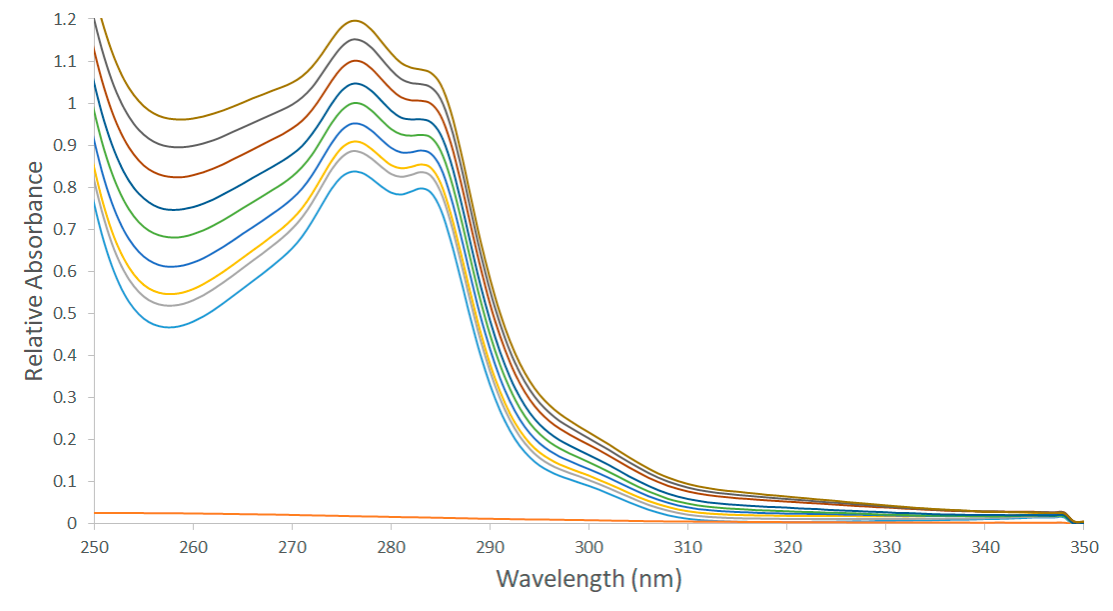
Absorbance spectra of 0.2 mM oxaliplatin, 0.2 mM *p*-SC4, and several mixtures containing increasing concentrations of oxaliplatin (0.00–0.21 mM) and a fixed concentration of 0.2 mM *p*-SC4; all in distilled water.

Oxaliplatin displayed weak absorbance through the studied wavelength range (Figure 2). While the spectrum of *p*-SC4 demonstrated significant absorption maxima at 277 and 284 nm. The spectra of the mixtures displayed a noticeable hyperchromic shift, which correlated with increasing the concentrations of oxaliplatin.

**Figure 3:**
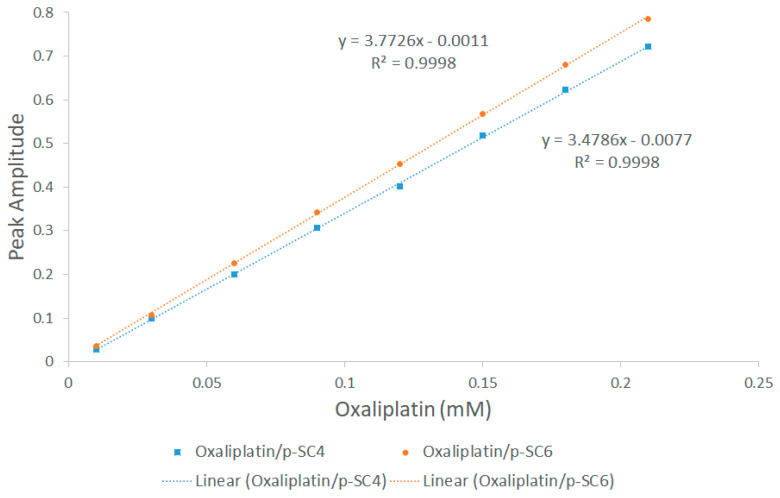
The plot of peak amplitudes at 267 nm obtained from the mixtures containing successively increasing concentrations of oxaliplatin (0.01–0.21 mM) and a fixed concentration of 0.2 mM *p*-SC4 and 0.2 mM *p*-SC6 all in distilled water using the spectrum of 0.2 mM of *p*-SC4 and *p*-SC6 as a divisor against the corresponding concentration of oxaliplatin in the complex mixtures.

The hyperchromic shift observed with increasing concentrations of oxaliplatin was investigated using our previously reported methods [31,66]. The zero-order spectra of the prepared mixtures were divided by the spectrum of *p*-SC4, and the first derivative of the ratio spectra was obtained using a scaling factor of 10 and Δλ = 4 nm [31,66]. The values of the peak amplitudes of the first derivative of the ratio spectra for the mixtures (Figure S2) were then obtained at 267 nm. Figure 3 shows a plot of the acquired peak amplitudes and the equivalent concentrations of oxaliplatin. Adding increasing concentrations of oxaliplatin to a fixed concentration of *p*-SC6 has exhibited similar behavior. A plot of the obtained peaks, in the case of oxaliplatin/*p*-SC6, and the corresponding concentrations of oxaliplatin is demonstrated in Figure 3. The nearly perfect linear relationships displayed in Figure 3 indicate that the hyperchromic shifts observed in the case of oxaliplatin*/p*-SC4 and oxaliplatin/*p*-SC4 are due to complexation between oxaliplatin and each of *p*-SC4 and *p*-SC6 (Figure 3).

**Figure 4:**
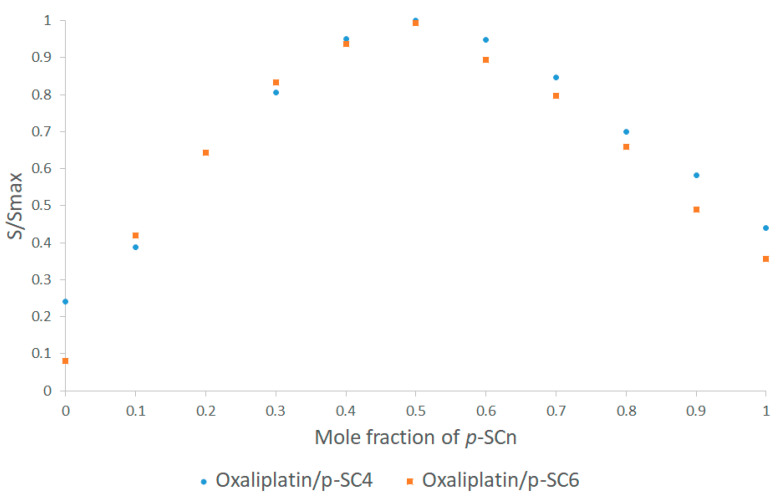
Normalized Job’s plot where each of the amplitude values, S, of the first derivative ratio spectra peaks were divided by the maximum amplitude, S_max_, are plotted against the mole fraction of the corresponding *p*-SC4 molar fraction and *p*-SC6 molar fraction.

The stoichiometry and the stability constants of the supramolecular complexes were studied using Job’s plot (the method of continuous variation) involving the derivative ratio method, using methods described previously [31,67,68,69]. From Job’s plots, it was revealed that the maximum amplitudes were observed at molar fractions of 0.5, indicating a host-guest complex stoichiometry of 1:1 for oxaliplatin/*p*-SC4 and oxaliplatin/*p*-SC6. A normalized form of these Job’s plots for both oxaliplatin/*p*-SC4 and oxaliplatin/*p*-SC6, where each of the amplitude values, S, were divided by the equivalent maximum amplitude, S_max_, are presented in Figure 4, respectively. The stability constants of the complexes were detected using methods detailed previously [31,70,71] to be 5.07 × 10^4^ M^−1^ and 6.3 × 10^4^ M^−1^ which correspond to complexation free energy of −6.39 and −6.52 kcal/mol, for oxaliplatin/*p*-SC4, and oxaliplatin/*p*-SC6, respectively. These values are within the range of the stability constants (0.01 × 10^3^–1.7 × 10^5^) M^−1^ previously reported for host-guest complexes, many of which are proposed for drug delivery, and many of them showed improved bioavailability, stability, and enhanced therapeutic effects [31,72,73,74,75,76,77,78,79,80,81,82,83].

### 3.3. In Vitro Cell Viability Assay

**Figure 5:**
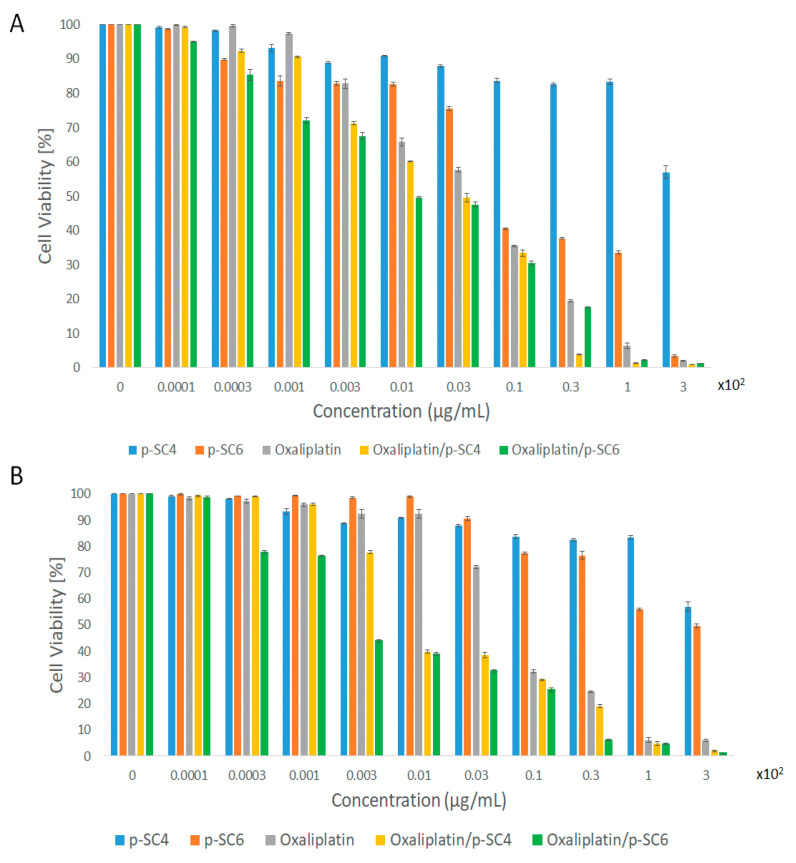
Evaluating the cytotoxicity of *p*-SC4, *p*-SC6, oxaliplatin, oxaliplatin/*p*-SC4, and oxaliplatin/*p*-SC6 at various concentrations ranging from 0.01 to 300 µg/mL using SRB assay in (**A**) HT-29 cells and (**B**) MCF-7 cells. An overall statistically significant decrease in cell viability was observed with oxaliplatin/*p*-SC4 and oxaliplatin/*p*-SC6 compared to free oxaliplatin (*p* < 0.05). Untreated cells were used as negative control and considered as 100%. All experiments were carried out in triplicates, and the mean values were calculated. Error bars represent ± standard deviation.

The anticancer activities of *p*-SC4, *p*-SC6, oxaliplatin, and the oxaliplatin complexed with each of *p*-SC4 and *p*-SC6 were assessed against HT-29 colorectal cancer cells and MCF-7 breast adenocarcinoma employing SRB assay (Figure 5). Both *p*-SC4 and *p*-SC6 were used as vehicle control, where *p*-SC4 showed no significant decrease in cell viability, while *p*-SC6 exhibited some cytotoxic effects. Our findings show that both complexes have significant in vitro anticancer activities against both involved cell lines compared to free oxaliplatin. The cytotoxic activities [IC_50_ in µg/mL, computed from concentration-response curves by Sigma Plot software, version 12.0 (System Software, San Jose, CA, USA), using an E-max model equation] of *p*-SC4, *p*-SC4, oxaliplatin, oxaliplatin/*p*-SC4, and oxaliplatin/*p*-SC6 against HT-29 and MCF-7 are presented in Table 2. The IC_50_ of both oxaliplatin/*p*-SCn complexes displayed almost twice that of the free drug against HT-29 cells, while the IC50 of oxaliplatin/*p*-SC4 and oxaliplatin/*p*-SC6 displayed about three times and eight times that of the free drug against MCF-7 cancer cells, respectively. The increased cytotoxic activity of both complexes compared to free oxaliplatin might be due to the enhancement of the water solubility, and hence bioavailability, of oxaliplatin upon complexation with *p*-SC4 and *p*-SC4 [84,85]. The improved solubility was further confirmed by the ADMET study conducted computationally and presented below. Additionally, it was demonstrated that calixarenes can selectively release the drugs in a controlled manner in cancer cells following a pH triggered mechanism. In other words, the encapsulated drug is released at a typical cancerous tissue’s pH [86]. Therefore, the formation of oxaliplatin/*p*-SCn complexes increases the selectivity of the drug towards cancer cells and can reduce the toxic effects against healthy cells. This highlights the impact of using *p*-SCn as a possible host molecule for different anticancer drugs.

These findings suggest using the developed complex for a possible decline of the therapeutic dose of oxaliplatin, which might be promising in reducing systemic toxic effects. Alternatively, clinicians may opt to use a higher dose of oxaliplatin in the developed complex to maximize cancer therapy. Additionally, these findings might lead to further in vivo investigations of the developed complex against not only colorectal cancer cells but also breast cancer.

### 3.4. Computational Results

#### 3.4.1. DFT Calculations

**Figure 6:**
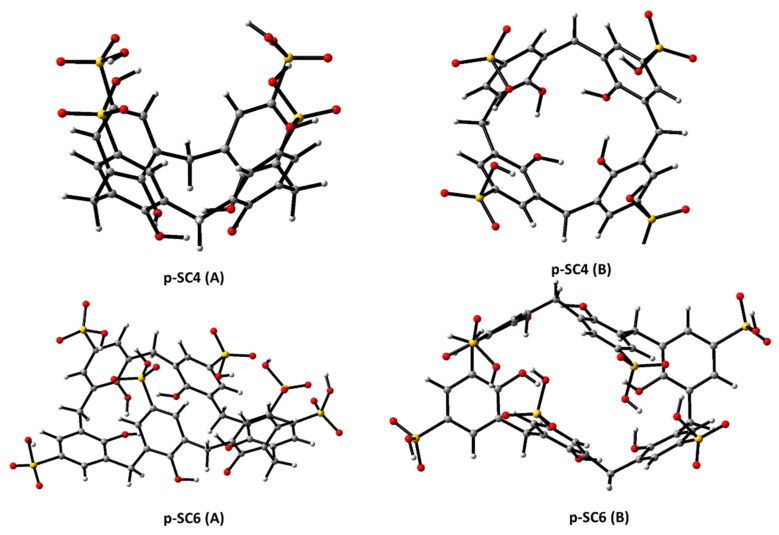
Optimized structures of *p*-SC4 and *p*-SC6 both in the side view (**A**) and top view (**B**). The 3D images of the optimized structures were prepared using GaussView visualization software [87].

The most stable structures of the *para*-sulfonatocalix[4]arene (*p*-SC4) and the *para*-sulfonatocalix[6]arene (*p*-SC6) are shown in Figure 6.

In the *p*-SC4 structure, characterized by the typical cone conformation and stabilized by intramolecular H-bonds in the lower rim between the OH groups and in the upper rim between the SO_3_H groups, the calculated distances for the upper and lower rim are respectively 7.139 Å and 3.791 Å. Such a conformation suggests a plausible entrance channel, through the upper rim, of a guest molecule. On the other hand, the investigated *p-*SC6 conformation is characterized by a less symmetric cavity. The hydrogen bonds established among the protonated sulfonate groups in the upper rim cause a strong distortion of the calixarene leading to its closure.

Recently it was shown that based on the volumes of both host and guest molecules, it is possible to predict the penetration of a guest into the cavity [33]. The calculated oxaliplatin volume is 186 Å^3^ and the potential insertion into the *p-*SC4 and *p-*SC6 cavities, which exhibit inner volumes of 626 Å^3^ and 989 Å^3^ respectively, could take place. However, several factors must be considered in the host-guest inclusion complexes formation.

**Scheme 1:**
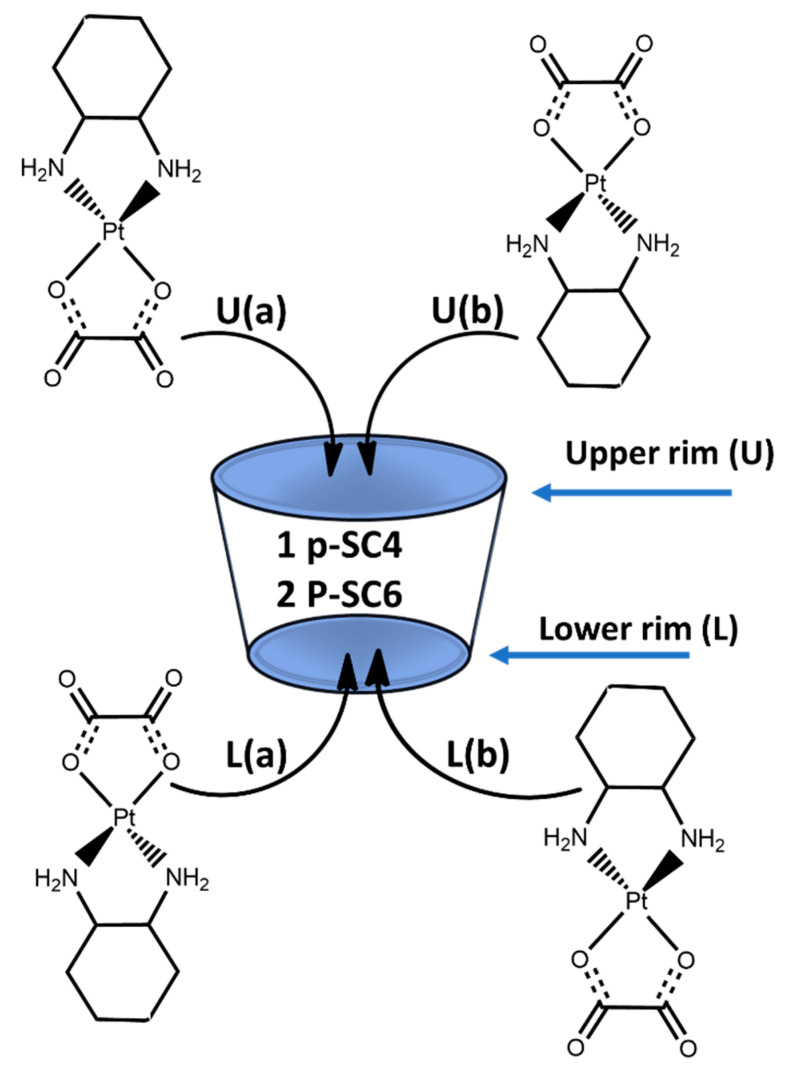
Four possible inclusion modes of oxaliplatin through the upper (U) and lower (L) rims of *p-*SC4 (1) and *p-*SC6 (2). In **U(a)** the oxalate ligand points inwards to the upper rim of the cavity and the cyclohexane points outwards. In **U(b)** the cyclohexane ligand points inwards to the upper rim of the cavity and the oxalate points outwards. In **L(a)** configuration the oxalate points inwards to the lower rim of the cavity and the cyclohexane points outwards. In **L(b)** configuration the cyclohexane points inwards to the lower rim of the cavity and the oxalate outwards.

Scheme 1 shows all the feasible modes of insertion, computationally examined, of the oxaliplatin drug into the *p*-SC4 and *p-*SC6 cavities. The oxaliplatin inclusion can occur from both the side of the upper rim (U) and the side of the lower rim (L). Additionally, the oxaliplatin can assume two different orientations during the insertion inside both the cavities. The arrangement in which the two oxygen atoms of the oxalate ligand point inwards to the cavity and the cyclohexane ring points outwards is labelled as (a), while in (b) arrangement the cyclohexane ring points inwards to the cavity and the oxalate points outwards.

**Figure 7:**
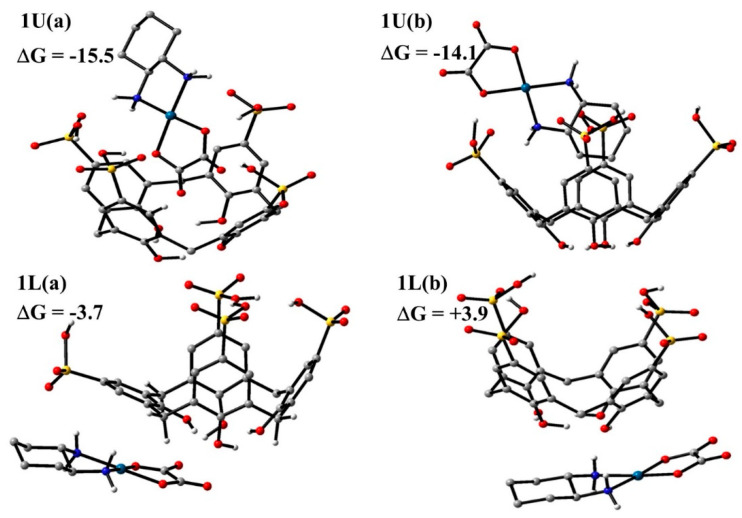
B97-D optimized geometrical structures of *p-*SC4 and binding energies (kcal/mol). The three-dimensional images of the optimized structures were prepared using GaussView 6.1.1 visualization software [87].

**Figure 8:**
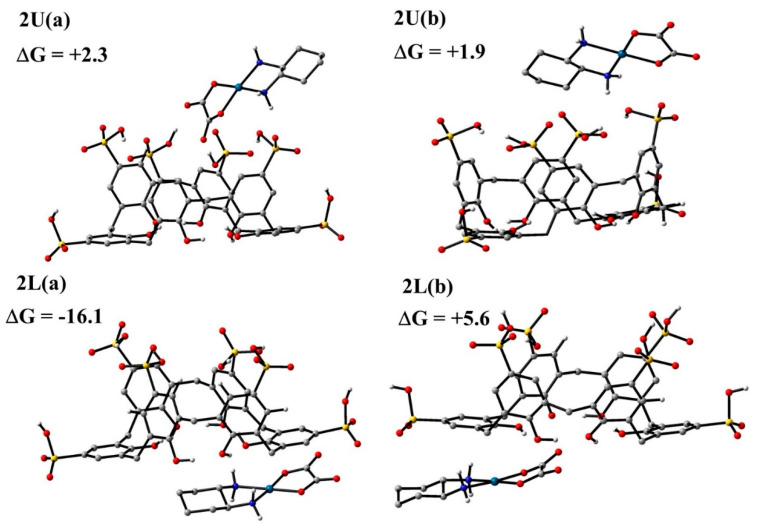
B97-D optimized geometrical structures of *p-*SC6 and binding energies (kcal/mol). The three-dimensional images of the optimized structures were prepared using GaussView 6.1.1 visualization software [87].

The fully optimized geometries of the host-guest complexes formed with *p-*SC4 and *p-*SC6, obtained without imposing any constraints, are reported in Figure 7 and Figure 8, respectively. The values of the calculated complexation free energies (including BSSE correction and entropy change corrections in solution) are reported in the same figures.

Regardless of the numerous attempts carried out adopting different reciprocal orientations of the oxaliplatin with respect to calixarenes, the guest always collapses into the reported structures shown in Figure 7 and Figure 8.

Looking at the host-guest complexes intercepted for the inclusion of oxaliplatin from the upper side in the *p-*SC4 host, the guest can assume both (a) and (b) orientations, obtaining type **1U(a)** and **1U(b)** conformations. In both the intercepted structures, the drug is included into the host cavity. In the conformation labeled as **1U(a)** the two oxygen atoms of the oxalate ligand point towards the interior of the calixarene from the upper rim and H-bond interactions with the protonated oxygen atom of the nearest sulfonate group are established. The cyclohexane ring points outwards, establishing minimal interactions with the host. In **1U(b)** insertion complex, the guest interacts with the cavity through the cyclohexane ligand and the oxalate group points outwards. This description of oxaliplatin insertion with its cyclohexane ring into the cavity mirrors the reported ^1^H NMR data.

For the inclusion from the lower side, instead, two association adducts indicated as **1L(a)** and **1L(b),** were found. In both the intercepted structures, the *p-*SC4 calixarene and the oxaliplatin complex weakly interact and the drug lies outside the macrocycle.

The calculated host-guest binding energy for the conformations **1U(a)**, **1U(b)**, **1L(a),** and **1L(b)** are −15.5, −14.1, −3.7, +3.9 kcal/mol, respectively.

Based on calculated insertion free energies, it appears that the structures in which the drug is included in the cavity, **1U(a)** and **1U(b)** configurations, are much more stable than those in which oxaliplatin does not form inclusion complexes, **1L(a)** and **1L(b)** configurations.

Definitely, except for the **1L(b)** arrangement, the guest interacts with both ends of the host, maximizing stabilizing forces. The **1L(b)** adduct is found to be 3.9 kcal/mol higher in energy with respect to the separated species. In that configuration, the guest is located under the host cavity with the hydrogen atoms of the two amino groups interacting with the oxygen atoms of the OH groups. The guest-host interactions destabilize the original host structure, changing the charge distribution, leading to a destabilization of the system.

These results agree very well with the experimental findings, which suggest that oxaliplatin is embedded within the cavity of *p*-SC4 and the computed complexation free energy has the same order of magnitude as that experimentally estimated, which corresponds to the value of −6.3 kcal mol^−1^. As it is reported in Figure 8, both (a) and (b) arrangements were taken into consideration for the inclusion of the drug from the lower side of the *p-*SC6 calixarene host, obtaining type **2U(a)** and **2U(b)** conformations. Likewise, for the inclusion from the lower side, oxaliplatin can assume both (a) and (b) orientations, and the systems identified as **2L(a)** and **2L(b)** were obtained.

In all the intercepted structures, oxaliplatin is located outside the host cavity. In the system having an (a) arrangement, hydrogen bonds between the oxalate group and the protonated sulfonate moiety (for **2U(a)** configuration) or OH groups (for **2L(a)** configuration) are formed. In both (b) arrangements, oxaliplatin prefers interacting with the cavity through the hydrogen atoms of the ammonia ligands.

In the **2L(a)** configuration, the interaction between oxaliplatin and the host cavity causes a significant deformation of the cavity, suggesting a stronger interaction of *p-*SC6 with the examined guest, compared to other intercepted adducts. The interaction-free energies computed for the structures **2U(a)**, **2U(b)**, **2L(a),** and **2L(b)** are −2.3, +1.9, −16.1, +5.6 kcal/mol, respectively.

In the **2L(a)** adduct, the guest interacts with the lower rim of the host, maximizing the stabilizing forces, and it is shown to possess the greatest complexation energy compared to the other examined complexes. Computational outcomes demonstrate that only the **2L(a)** adduct may be plausible when the oxaliplatin interacts with the *p*-SC6 calixarene host and H-bonds and other weak interactions determine its stability. Furthermore, the order of stability calculated for the complexation between oxaliplatin and *p-*SC4 or *p-*SC6 is in agreement with that observed experimentally.

#### 3.4.2. ADMET Studies

The ADMET study was also performed using Discovery Studio 4.0 to predict the biological properties of the most stable complexes obtained, namely 1U (b) and 2L (a). The results obtained (Table S1; Figures S3 and S4) support the improvement of oxaliplatin’s biological properties upon its complexation with each of *p*-SC4 and *p*-SC6. The absorption of oxaliplatin is predicted to be better than both complexes, and hence it will be absorbed by the cancer cells without the carrier; thus, better efficacy will be obtained. The passage through BBB is expected to be decreased in the case of both complexes and hence there would be lower toxicity to brain cells. Additionally, binding of complexes to plasma protein is better than that of free oxaliplatin and hence there is expected to be prolonged action of the drug.

## 4. Conclusions

Findings from ^1^HNMR, UV spectroscopy, Job’s plot, and theoretical calculations support successful complexation between oxaliplatin and each of *p*-SC4 and *p*-SC6 in a 1:1 molar ratio. The stability constants of oxaliplatin/*p*-SC4 and oxaliplatin/*p*-SC6 complexes were calculated to be 5.07 × 10^4^ M^−1^ and 6.3 × 10^4^ M^−1^, which corresponds to the free energy of complexation of −6.39 and −6.52 kcal/mol, respectively. The stability of both complexes in aqueous solutions suggests its use as a possible drug delivery vehicle. Both ^1^H NMR spectroscopy and DFT calculations indicate that oxaliplatin was embedded within the cavity of *p*-SC4 while in the formed complex with *p*-SC6; the anticancer drug was not fully included within the host cavity but was strongly stabilized by hydrogen bonds and other weak interactions.

Data from the SRB assay showed that the IC_50_ of oxaliplatin/*p*-SC4 and oxaliplatin/*p*-SC6 complexes was almost twice that of the free oxaliplatin against HT-29 cells (IC_50_2, 1.3 and 3.9 µg/mL, respectively). Additionally, the IC_50_ of oxaliplatin/*p*-SC4 and oxaliplatin/*p*-SC6 complexes was three and eight times that of the free oxaliplatin against MCF-7 breast adenocarcinoma (IC_50_ 5, 1.56, and 0.59 µg/mL, respectively). Besides, ADMET computational investigation demonstrated the improvement of the biological properties of oxaliplatin upon its complexation with each of *p*-SC4 and *p*-SC6. These findings support the potential use of the proposed complexes for chemotherapy of cancer patients, using oxaliplatin to either reduce systemic toxic effects or increase the oxaliplatin dose (at similar side effects to that of the free drug) to maximize its therapeutic impact.

## Figures and Tables

**Table 1:** Detected shielding (ppm) for allocated protons in oxaliplatin, *p-*SC4, and 1:1 M ratio mixture in D_2_O. Proton labels are shown in Figure S1.

Proton Signals	Individual Molecules	1:1 Molar Ratio
3ax	0.92–1.00	0.19–0.25
2ax	1.16–1.25	0.38–0.44
3eq	1.39–1.46	0.98–1.04
2eq	1.81–1.88	1.16–1.21
1	2.03–2.04	1.63–1.66
Hy	3.67	3.83

Protons 3ax, 2ax, 3eq, 2eq, and 1 are in oxaliplatin and Hy is in p-SC4.

**Table 2:** In vitro anticancer activities of *p*-SC4, *p*-SC6, oxaliplatin, and the oxaliplatin complexed with each of *p*-SC4 and *p*-SC6 against two different cancer cell lines.

Cells	In Vitro Anticancer Activity (IC50 in µg/mL)				
	*p*-SC4	*p*-SC6	Oxaliplatin	Oxaliplatin/*p*-SC4	Oxaliplatin/*p*-SC6
HT-29	N/A	10	3.9	2	1.3
MCF-7	N/A	220	5	1.56	0.59

## Supplementary Materials

Figure S1: ^1^HNMR spectra of (A) *p*-SC4 alone, (B) oxaliplatin alone, and (C) equilmolar ratio of amount of oxaliplatin and *p*-SC4. Structures of oxaliplatin and *p*-SC4 are shown with appropriate protons labeled, Figure S2: First derivative of ratio spectra of the mixtures containing successively increasing concentrations of oxaliplatin (ranging from 0.01-0.21 mM) and a fixed concentration of 0.2 mM *p*-SC4 all in distilled water using the spectrum of 0.2 mM of *p*-SC4 as a divisor, Table S1: ADMET study rsults of oxaliplatin, oxaliplatin/*p*-SC4, and oxaliplatin/*p*-SC6 complexes as performed by Discovery Studio 4.0. ADMET values and descriptors are provided below, Figure S3: The shift in the biological properties between oxaliplatin alone and oxaliplatin/p-SC4 complex, Figure S4: The shift in the biological properties between oxaliplatin alone and oxaliplatin in complex with SC6-calixarene.
