# Supplementary material for: Host-Guest Complexation of Oxaliplatin and Para-Sulfonatocalix[n]Arenes for Potential Use in Cancer Therapy
Source: Molecules. 2020 Dec 14;25(24):5926. doi: 10.3390/molecules25245926 (PMC7765097; doi:10.3390/molecules25245926)
Supplement: Supplementary file 1 [file molecules-25-05926-s001.pdf]

Electronic Supplementary Information

Host-guest complexation of oxaliplatin and *para*-sulfonatocalix[n]arenes for potential use in cancer therapy

Sherif Ashraf Fahmy[1], Fortuna Ponte[2], Iten M. Fawzy[3], Emilia Sicilia[2], Udo Bakowsky[4]* and Hassan M. E. Azzazy[1]*

[1]Department of Chemistry, School of Sciences & Engineering, The American University in Cairo, AUC Avenue, PO. Box 74, New Cairo 11835, Egypt; sheriffahmy@aucegypt.edu

[2] Department of Chemistry and Chemical Technologies, University of Calabria, Arcavacata di Rende, 87036, Italy

[3]Pharmaceutical Chemistry Department, Faculty of Pharmaceutical Sciences and Pharmaceutical Industries, Future University in Egypt, Cairo 12311, Egypt; iten.mamdouh@fue.edu.eg

[4] Department of Pharmaceutics and Biopharmaceutics, University of Marburg, Robert- Koch-Str. 4, 35037 Marburg, Germany.

* Correspondence: ubakowsky@aol.com (U.B.); hazzazy@aucegypt.edu (H.M.E.-S.A.);
Tel.: +49-(0)-6421-28-2-58-84 (U.B.); +2 02 2615 2559; Fax: +2 02 2795 7565 (H.M.E.-S.A.)

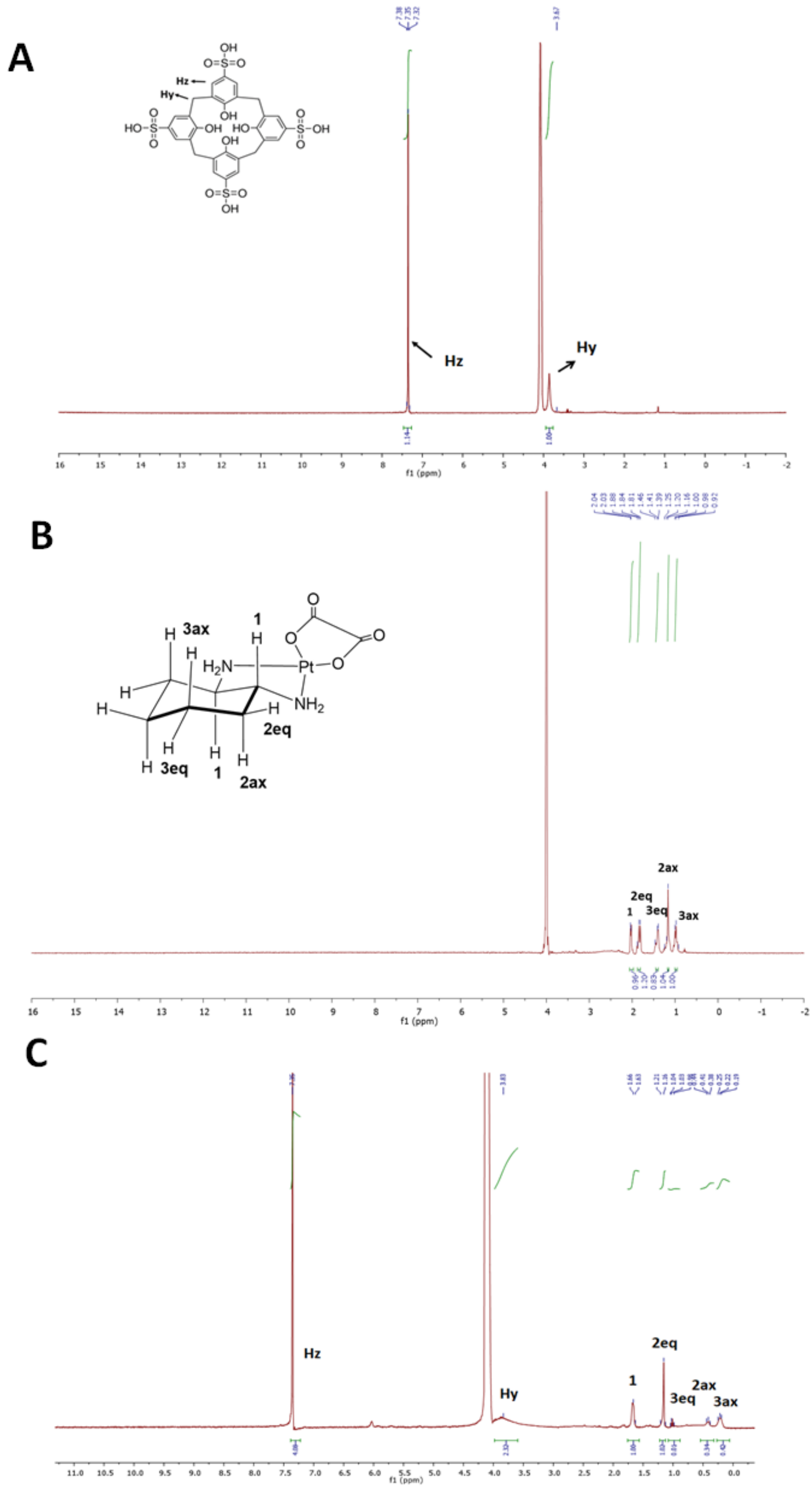
A
Hz
Hy
f1 (ppm)
B
3ax
1
2eq
3eq
2ax
f1 (ppm)
C
Hz
Hy
1
2eq
3eq
2ax
3ax
f1 (ppm)

Figure S1. ^{1}HNMR spectra of (A) *p*-SC4 alone, (B) oxaliplatin alone, and (C) equilmolar ratio of amount of oxaliplatin and *p*-SC4. Structures of oxaliplatin and *p*-SC4 are shown with appropriate protons labeled.

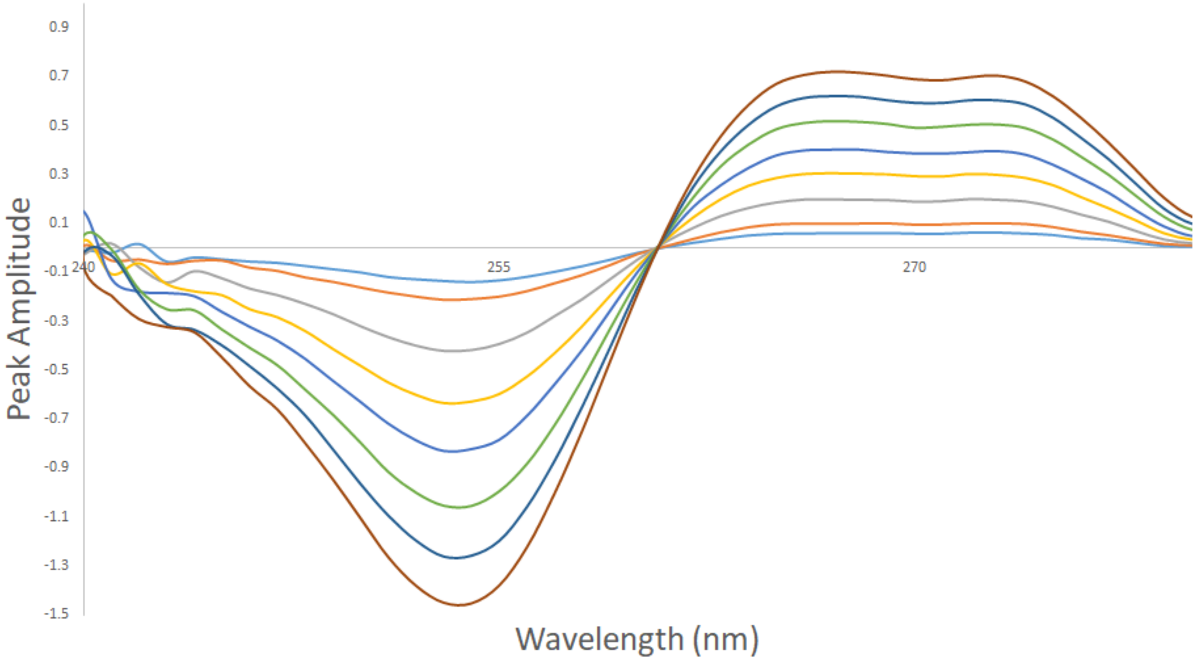

Figure S2 First derivative of ratio spectra of the mixtures containing successively increasing concentrations of oxaliplatin (ranging from 0.01-0.21 mM) and a fixed concentration of 0.2 mM *p*-SC4 all in distilled water using the spectrum of 0.2 mM of *p*-SC4 as a divisor.

Table S1. ADMET study rsults of oxaliplatin, oxaliplatin/*p*-SC4, and oxaliplatin/*p*-SC6 complexes as performed by Discovery Studio 4.0.

ADMET values and descriptors are provided below.

	Aqueous Solubility Level	Human Intestinal Absorption	Hepatotoxicity	CYP-2D6	BBB (Blood Brain Barrier Penetration Level)	PPB (Plasma Protein Binding)
**Oxaliplatin**	3	0	1	0	3	0
**Oxaliplatin/*p*-SC4**	5	3	1	0	4	1
**Oxaliplatin/*p*-SC6**	5	3	1	0	4	1

Discovery Studio 4.0 ADMET values and descriptors

Discovery Studio 4.0 Descriptors	Aqueous Solubility Level			Human Intestinal Absorption		Hepatotoxicity		CYP-2D6		BBB (Blood Brain Barrier Penetration Level)		PPB (Plasma Protein Binding)	
	Level	***Value***	***Description***	***Level***	***Description***	***Level***	***Description***	***Level***	***Description***	***Level***	***Description***	***Level***	***Description***
	0	log (molar solubility) < −8.0	Extremely low	0	Good absorption	0	Non-toxic	0	Non-inhibitor	0	Very High	0	Binding is <90%
	1	−8.0 < log (molar solubility) < −6.0	No, very low, but possible										
	2	−6.0 < log (molar solubility) < −4.0	Yes, low	1	Moderate absorption					1	High		
	3	−4.0 < log (molar solubility) < −2.0	Yes, good							2	Medium	1	Binding is ≥90%
	4	−2.0 < log (molar solubility) < 0.0	Yes, optimal	2	Low absorption	1	Toxic	1	Inhibitor	3	Low		
	5	0.0 < log (molar solubility)	No, too soluble							4	Undefined		
	6	−1000	Warning: molecules with one or more unknown AlogP98 types	3	Very Low absorption					5	Warning: molecules with one or more unknown AlogP calculation	2	Binding is ≥95%

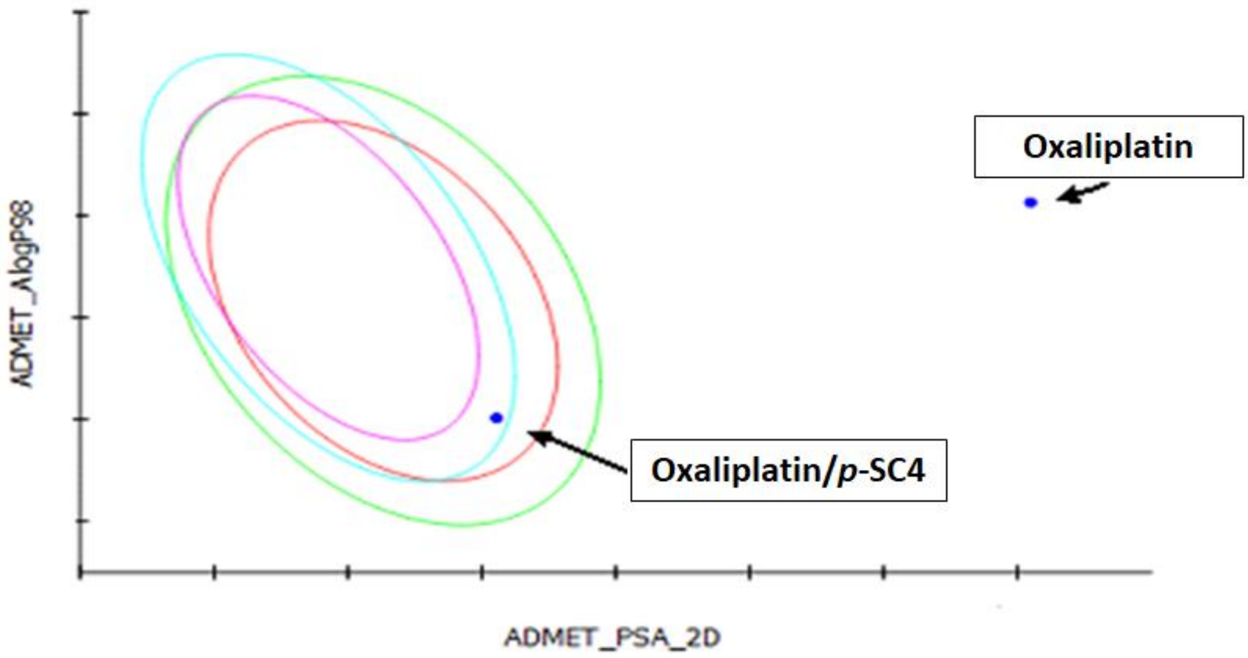

Figure S3. The shift in the biological properties between oxaliplatin alone and oxaliplatin/p-SC4 complex.

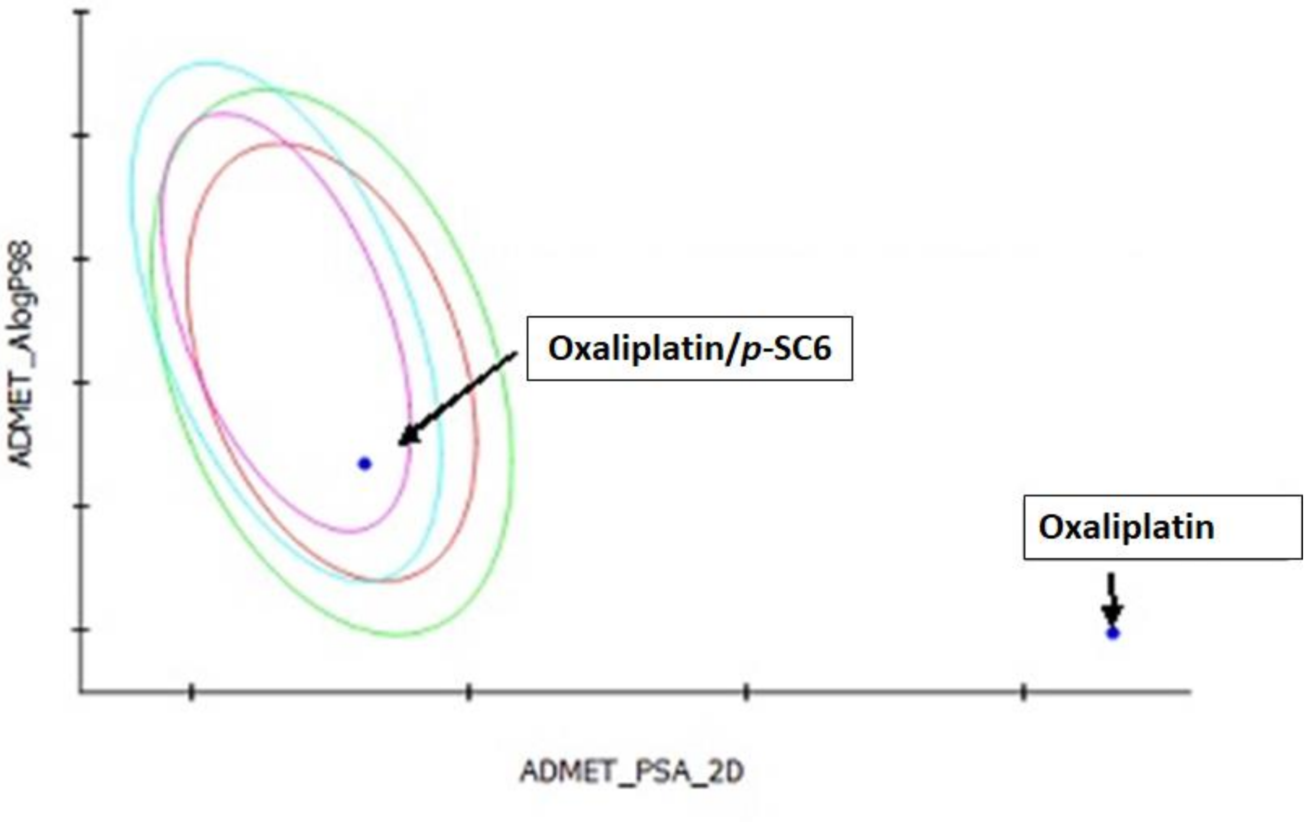

Figure S4. The shift in the biological properties between oxaliplatin alone and oxaliplatin in complex with SC6-calixarene.
